# Protective effect of standardised fruit extract of *Garcinia cowa* Roxb. ex Choisy against ethanol induced gastric mucosal lesions in Wistar rats

**DOI:** 10.1080/07853890.2021.1981548

**Published:** 2021-09-23

**Authors:** Prakash Chandra Gupta, Ashish Kar, Nisha Sharma, Prashant Kumar Singh, Naba Kumar Goswami, Satyanshu Kumar

**Affiliations:** a University Institute of Pharmacy, Chhatrapati Shahu Ji Maharaj University, Kanpur, India; b Northeastern Regional Centre, The Energy and Resources Institute (TERI), Guwahati, India; c ICAR-Directorate of Medicinal and Aromatic Plants Research, Boriavi, Anand, India

**Keywords:** Ethanol, gastric lesion, malondialdehyde, antioxidant, *Garcinia cowa*

## Abstract

**Background and aim:**

The fruit of *Garcinia* is a rich and valuable source of bioactive compounds and is traditionally used for treating wounds and ulcers. The present study was carried out to investigate the protective effect of chromatographically standardized fruit extract of *Garcinia cowa* (GCE) on ethanol-induced gastric lesions in rats and its possible mechanisms.

**Methods:**

The effect of GCE (200 and 400 mg/kg body weight) was evaluated by determining various gastric ulcer parameters like gastric wall mucus, non-protein sulfhydryls (NP-SH) content, microvascular permeability, endogenous antioxidant enzyme, and gastric histopathological study.

**Results and conclusions:**

Oral administration of GCE at doses of 200 and 400 mg/kg exhibited significant (*p* < .01) dose-dependent inhibition of ulcer index by 18.94–44.02%, respectively. Pre-treatment of rats with GCE (400 mg/kg) significantly restored the depleted gastric wall mucus level by 34.09% and NP-SH content by 33.35% induced by ethanol administration. In addition, GCE (400 mg/kg) showed a significant decrease in microvascular permeability of Evans Blue by 47.43%, rationalizing its protective effect. Furthermore, a significant increase in oxidative enzyme levels with reduction in malondialdehyde level and elevation of superoxide dismutase (SOD) activity was observed in the GCE treated group as compared to the ulcer control group. The histopathological assessment also confirmed the protective nature of GCE. HPTLC analysis showed the presence of 0.27%, 0.11% w/w gallic acid, and amentoflavone, respectively in GCE. The content of α-mangostin and xanthochymol in the *G. cowa* extract sample quantified by HPLC-PDA method was 0.72 and 8.46%, respectively. The results obtained indicate that the protective effect of GCE against gastric ulcers in rats through multiple actions confirmed by the reduction of oxidative stress and restoration of adhered gastric mucus, NP-SH content, and histological architecture.

**KEY MESSAGES**
- Ethanol is the most typical ulcerogenic agent and has been shown to extend the risk of ulcer in humans.
- Natural products are promising alternative medication for the development of new drugs to regulate gastrointestinal diseases.
- *Garcinia cowa* protects the gastric mucosa through multiple actions that include restoration of adhered gastric mucus and inhibition of lipid peroxidation.

## 1. Introduction

A gastric ulcer is a benign lesion of the gastric mucosa that influences about 10% of the planet population [1]. Peptic ulcer disease has multi-factorial pathophysiology that is caused by an imbalance between protective factors of the gastric mucosa, such as adequate mucus, blood flow, and bicarbonate secretion, prostaglandin, sulfhydryl (SH) compounds, and antioxidant enzymes, and aggressive factors like acid and pepsin [2]. Factors that may increase the incidence of peptic ulcer disease include alcohol consumption, wide use of non-steroidal anti-inflammatory drugs (NSAIDs), altered prostaglandin E series metabolism, stressful lifestyle, and bad dietary habits [3,4]. *Helicobacter pylori* infection is also one of the major causes of peptic ulcer disease, chronic gastritis, and gastric adenocarcinoma [5].

Ethanol is a most typical ulcerogenic agent and has been shown to extend the risk of ulcer in humans and has been evident to produce potent ulceration in rats [6]. The integrity of the stomach mucosal barrier is disrupted by ethanol due to cell exfoliation, thus increasing mucosal permeability and, in rare cases, causing bleeding [7].

Despite the availability of several medications for the treatment of gastric ulcers, such as H_2_ receptor antagonists and proton pump inhibitors, there is currently no comprehensive cure for the illness. The development of tolerance, the rate of relapses, adverse effects, and drug interactions were all observed in clinical trials of these medicines [8,9] (Table 1).

**Table 1.** List of drugs commonly used for gastric ulceration therapy with side effects and drug interaction.

Name of drug	Side effects	Drug interaction
Cimetidine	Cancer occurrence, Bradycardia, Psychosis, acute pancreatitis, etc.	Phenytoin, warfarin, Theophylline, Analgesics, etc.
Ranitidine	Hepatitis, cardiac arrest, delirium, bone marrow aplasia, etc.	Nifedipine, Ketoconazole, quinolones, etc.
Omeprazole	Enteric infection, sustained achlorhydria, renal failure, chronic constipation, etc.	Phenytoin, warfarin, diazepam, etc.
Antacids	Osteomalacia, Hyperaluminaemia, etc.	Allopurinol, iron, norfloxacin, etc.
Sucralfate	Osteomalacia, hypophosphataemic Pneumonia, oesophageal bezoars	Cimetidine, thyroxin, norfloxacin, ciprofloxacin, etc.
Pirenzipine	Increased heart rate in ventilated patients	Amantadine, antidepressants, etc.
Misoprostol	Colic, diarrhoea, miscarriage, uterine bleeding	Indomethacin, ibuprofen, chlorpropamide, paracetamol, etc.

Most of the anti-secretary medications decrease acid output, thus providing rapid symptom relief, but there have been reports of long-term side effects and relapses. Natural medicines, on the other hand, primarily augment defensive factors and may be sluggish to effect, but are reliable and safe. Several natural medicines have been reported to have anti-ulcer activity due to their primary effect on mucosal defensive factors [10,11]. Furthermore, herbal treatment for gastric ulcer costs roughly one-sixth that of Western medicine [12]. As a result, natural medicines, whether used alone or in combination with other pharmaceuticals, should be thoroughly investigated.

Borrelli and Izzo [13] reported a wide range of chemical compounds with antiulcer action that was extracted from medicinal plants. This is a compelling reason to explore the antiulcer properties of medicinal herbs with a long history of usage in stomach disorders. As a result, medicinal plants and their products may be a better alternative therapy that has lesser adverse effects with low cost and the ability to scavenge free radicals [14,15]. Medicinal plants have been thoroughly studied in the literature as a viable alternative treatment for the development of novel medications to regulate gastrointestinal disorders [16,17]. The genus *Garcinia*, belonging to the family Clusiaceae is a polygamous tree that has been widely investigated for different biological activities like cytotoxic, antimicrobial, free radical scavenging activity [18]. *Garcinia cowa* Roxb. Choisy provincially known as Kuji-Thekera, is widely found in South East Asia and Africa [19,20]. Different parts of this plant are used as traditional folk medicine. *Garcinia* fruit infusions have been used in traditional medicine to heal wounds, ulcers, and dysentery [21]. The fruits of the plant have been used for indigestion, enhancement of blood circulation and as expectorant while root and bark have been used in the treatment of fever [22,23]. Various parts of the plant have been explored for its phytoconstituents and biological activities. *G. cowa* is reported to possess antitumor [24], antimalarial [25], antimicrobial [26], anticancer [27], antioxidant [22], anti-inflammatory [22], and antiplatelet [28] activities.

*G. cowa* is a rich source of secondary metabolites and various chemical constituents have been isolated notably xanthones, benzophenone, biphenyl, and bioflavonoid [23,29]. The bioactive compound, such as morelloflavone, daucosterol, p-coumaric acid [30], cowaxanthone, cowanin, α-mangostin [31], cambogin, guttiferone K [32] have been isolated from *G. cowa* fruits. Based on the traditional uses, the present study was done to evaluate the antiulcerogenic activity of *G. cowa* fruit against ethanol-induced gastric injury in rats. Acute toxicity testing is the initial step in determining the safety of a drug, and it includes rapid procedures to determine the concentration that causes harm to the test organisms. As a result, we conducted an acute toxicity study to determine the safety of *G. cowa*.

## 2. Material and methods

### 2.1. Chemicals and reagents

Alcian blue, aluminium chloride, Folin-Ciocalteu reagent, ethylene diamine tetra acetic acid (EDTA), 5,5-dithio-bis-(2-nitrobenzoic acid (DTNB), gallic acid, thiobarbituric acid, trichloroacetic acid, toluene, ethyl acetate, formic acid, formaldehyde, sodium hydroxide, rutin, phosphoric acid, pyrogallol, haematoxylin, eosin were purchased from HiMedia Laboratories Pvt Ltd, Mumbai, India, and HPTLC plates, tris-HCl buffer, ethanol, amentoflavone were purchased from Sigma–Aldrich Chemical Co. (St. Louis, MO, USA).

### 2.2. Collection and identification of plant material

Fresh fruits of *G. cowa* were collected from Bherakuchi pothar village, Kamrup Metro District (26.05′52.53″ N, 92.07′51.05″ E), Assam, India in the month of August 2018. The plant specimen was identified, authenticated by Dr. Dilip Roy, Taxonomist, Botanical Survey of India, Shillong, India, and voucher specimen (FR-GC/A.Kar-15) was deposited in herbarium section of TERI-Guwahati for future reference.

### 2.3. Preparation of extract

The collected fresh fruits of *G. cowa* were cut into small pieces. The pulp of the fruits was air-dried under the controlled conditions and ground to the powder before extraction. Powdered air-dried material (400 g) was soaked in 70% ethanol (2.5 *L* × 4 times) for 72 h followed by filtration done twice through a muslin cloth and Whatman no. 1 filter paper simultaneously. The procedure was repeated three times and all the filtrates were combined. The combined filtrate was evaporated in a rotary evaporator (Buchi, New Castle, DE, USA) (40 °C) under reduced pressure and lyophilized (Ratan Raj Co., India) to get the hydroalcoholic extract of *G. cowa* (GCE) with a yield of 7.4% w/v.

### 2.4. Physicochemical standardization and HPTLC finger printing

The powdered fruit pulps were standardized as per pharmacopoeial procedures. The hydroalcoholic extract of the fruit pulp of *G. cowa* was detected for the presence of major phytochemical constituents. Based on the presence of phytochemical classes, HPTLC analysis was done to quantify the phenolic and flavonoid compounds. HPTLC analysis of GCE was performed on pre-activated (120 °C) silica gel 60 F_254_ HPTLC plates along with gallic acid and amentoflavone. The dried TLC plate was then eluted in solvent system toulene: ethyl acetate: formic acid (8:2:1). After elution, the silica gel plates were dried and densitometrically scanned at the wavelength of 366 nm for estimation of gallic acid and amentoflavone (WinCats software, CAMAG, Muttenz, Switzerland). The percentage of gallic acid and amentoflavone present in the extract was calculated by calibration using peak height ratio.

### 2.5. Characterization of bioactive compounds by HPLC

#### 2.5.1. Preparation of standards solutions and sample

The standard stock solutions (1 mg/ml) of α-mangostin and xanthochymol were prepared in HPLC grade methanol and stored in a refrigerator at 4 °C. Working solutions of lower concentrations of reference compounds were prepared by appropriate dilution of the stock solutions in methanol. The stock solution of GCE was prepared in methanol (1 mg/ml). The solutions of standards and extract were filtered through a 0.22 μm membrane filter before use in HPLC-PDA analysis.

#### 2.5.2. Chromatographic conditions

Chromatographic separation was achieved using an HPLC (Waters, Milford, MA, USA) system consisting of quaternary pumps, an in-line vacuum degasser, and a photodiode array detector (Waters 2996, Waters, Milford, MA, USA). The instrumentation was controlled by using Empower 3.0 software (Waters). Separation of α-mangostin and xanthochymol was achieved on a C-18 XBridge^®^ column (250 × 4.6 mm, 5.0 µm, Waters) in an isocratic elution mode. The mixture of acetonitrile-water (9:1, solvent A, 30%) and methanol with acetic acid (0.5%, solvent B, 70%) as mobile phase, with a flow rate of 0.5 ml/min, injection volume of 20 µl, with total run time of 35 min was selected to ensure any late eluting peak. The peaks obtained in the chromatograms of extracts were monitored in the wavelength range of 200–350 nm using a PDA detector. However, the wavelength selected for quantitative analysis of two analytes in GCE was 258 nm.

#### 2.5.3. Quantification of α-mangostin and xanthochymol in G. cowa extract

The identity of the peaks in the extract samples was confirmed by spiking a known concentration of the standards in extract samples and determination of retention time, matching PDA spectra and changes in the peak area. The concentration of α-mangostin and xanthochymol in GCE was calculated using the peak area by linear regression equations.

### 2.6. Estimation of total phenolic content

The Folin-Ciocalteu method was used to calculate the total phenolic content (TPC) of GCE [33]. In a nutshell, 0.5 ml GCE (1.0 mg/ml) was well mixed with 2.5 ml of 0.2 N Folin-Ciocalteu reagent for 5 min before adding 2.0 ml of Na_2_CO_3_ (75 g/l). Resulting mixture was then incubated for 2 h at 37 °C. Theabsorbance was measured at 760 nm by UV spectrophotometer. The quantity of phenolic content in the extract was expressed as milligrams of gallic acid per gram equivalent (GAE).

### 2.7. Estimation of total flavonoid content

The colorimetric method was used to determine the total flavonoid content (TFC) of GCE [34]. In a nutshell, 0.5 ml GCE was combined with 2.0 ml distilled water before being added to 0.15 ml of a 5% NaNO_2_ solution. After 6 min of incubation, 0.15 ml of 10% AlCl_3_ solution was added and left to stand for 6 min before being followed by the addition of 2.0 ml of 4% sodium hydroxide solution to the mixture. The sample was immediately diluted with water to reach a total volume of 5.0 ml, and the mixture was thoroughly mixed before being left aside for another 15 min. At a wavelength of 510 nm, the absorbance of the mixture was measured. The total flavonoid content was expressed as equivalent to rutin in mg/g of the extracts.

### 2.8. Experimental animals

Thirty Wistar rats (160–180 g) of either sex and 15 female Swiss albino mice (30–35 g) were purchased from Laboratory Animal House Facility, CSIR-Indian Institute of Toxicology Research, Lucknow, India. All the animals were acclimatized for at least one week in the animal house under standard conditions of a temperature of (25 ± 2 °C), relative humidity 45–55%, and 12 h light/dark cycle. The animals were fed with standard rodent feed (Ashirwad, India) and water. The rats were randomly assigned to different experimental groups, each containing six rats. Before inducing ulcers, the animals have fasted for 24 h though the water was allowed *ad libitum.* All the animal experiments were conducted in compliance with the CPCSEA guidelines for the care and use of experimental animals and with prior permission from the Institutional Animal Ethics Committee of University Institute of Pharmacy, Chhatrapati Shahu Ji Maharaj University (CSJMU), Kanpur (1589/GO/Re/S/2012/CPCSEA).

### 2.9. Acute toxicity study

Acute toxicity study of GCE was performed according to the up and down procedure for dose selection in accordance with OECD tests guidelines No. 425 [35]. Female Swiss albino mice were randomly selected for the study. Mice were divided into three groups of five mice each. Group I served as the control and the other two groups were sequentially treated with test drugs. GCE was administered orally to the overnight fasted mice. Animals of the control group received 1% CMC whereas the treated groups received a single oral dose of 2000 and 5000 mg/kg body weight. After dosing, food was withheld for 3–4 h while animals were closely observed for any toxic effect within the first 6 h and then at regular intervals for a total period of 14 days. Surviving mice were observed to determine the onset of toxic reactions.

### 2.10. Pharmacological evaluation

#### 2.10.1. Drug treatment protocol

All the standard and tested drugs were suspended in 1% carboxymethyl cellulose (CMC) and administered orally (by gavage). Test drug (GCE) in doses of 200 mg/kg b.w. (morning and evening with total 400 mg/kg b.w./day) and 400 mg/kg b.w. (morning and evening with total 800 mg/kg b.w./day) and standard drug H_2_ receptor blocker ranitidine (RAN) in the dose of 50 mg/kg b.w. (morning and evening with total 100 mg/kg b.w./day) were administered orally with the help of an orogastric tube twice daily for 5 days for acute ulcer protective studies in ethanol-induced ulcer model. The selected doses of GCE were determined based on the acute oral toxicity test discussed under paragraph 3.4. The dose of ranitidine was selected based on the well-established dose-response curve previously published and reported studies [36]. The Control group of animals received 1% carboxymethyl cellulose in distilled water.

#### 2.10.2. Ethanol (EtOH)-induced ulcers

After 5 days of treatment, animals have fasted for 24 h and care was taken to avoid coprophagy. Absolute ethanol was used as an ulcerogenic agent at a dose of 1 ml/200 g body weight of rat [37]. One hour after the oral administration of ethanol, the animals were killed by cervical dislocation. The stomach was removed surgically and opened along the greater curvature, rinsed with saline water (0.9%), and then mucosa was exposed for macroscopic evaluation. The number of ulcers was noted and recorded for the severity [38]. The ulcer index (UI) and percent protection were calculated by using the equation

$$UI=\frac{U_{N}+\;U_{S}+U_{P}}{10}\text{ and \% }\mathrm{protection}=\;[\frac{{UI}_{C}+\;{UI}_{T}\;}{{UI}_{C}}\;x\;100],$$

wherein *U_N_*, *U_S_*, *U_P_*, *UI_C_*, and *UI_T_* are the average number of ulcers per animal, an average of severity score, percentage of animals with the ulcer, *UI* of the control group, and *UI* of the treated group, respectively [39].

A small portion of the stomach was taken and kept in a 10% formalin solution for histopathological study. The remaining portion of the stomach after weighing was subsequently homogenized in phosphate buffer (pH 7.4) for the study of antioxidant parameters, such as lipid peroxidation and superoxide dismutase. The homogenized buffer solution was kept in a deep freezer at −40 °C until further analysis. The pH of gastric juice was also determined by digital pH metre (Elico, Hyderabad, India). The gastric content of the stomachs was drained into centrifuge tubes, subsequently centrifuged for 10 min at 3000 g, and the supernatant was assessed for pH measurement [40].

#### 2.10.3. Estimation of gastric wall mucus

Gastric wall mucus was determined according to the method of Corne et al. [41]. The glandular segments from the stomach were removed, weighed, and soaked for 2 h in a tube containing 1% Alcian blue solution (0.16 M sucrose in 0.05 M sodium acetate, pH 5.8). Then Alcian blue complexed with mucus was centrifuged and the absorbency of supernatant was measured at 498 nm. The quantity of mucus was calculated by the standard calibration curve of Alcian blue and the result was expressed in µg/g of glandular tissue.

#### 2.10.4. Estimation of non-protein sulfhydryl groups (NP–SH)

NP-SH content of gastric mucosa was estimated according to the method of Sedlak and Lindsay [42]. The glandular portion of the rat stomach was homogenized in ice-cold 0.02 M ethylene diamine tetra-acetic acid (EDTA). Aliquots of 5.0 ml of the homogenates were mixed in 15 ml test tubes with 4 ml distilled water and 1.0 ml 50% trichloroacetic acid. The tubes were centrifuged at 3000 g after being shaken intermittently for 10–15 min. The sample was shaken after an aliquot of 2.0 ml supernatant was combined with 4.0 ml of 0.4 M Tris buffer, pH 8.9, and 0.1 ml of 0.4 percent DTNB [5,5-dithio-bis-(2-nitrobenzoic acid)]. The absorbance was measured at 412 nm within 5 min of adding DTNB against a reagent blank with no homogenate.

#### 2.10.5. Determination of gastric mucosal microvascular permeability

Gastric microvascular permeability was evaluated on 1 h after ethanol treatment by measuring the extravasated amount of Evans blue dye (EBD) in mucosa as an indicator for increased capillary permeability. EBD (10 mg/kg) was injected intravenously for 30 min before the animal killing. The gastric mucosa was scraped from the stomach and soaked overnight in 1 ml 1 N KOH at 37 °C. Then 9 ml of a mixed solution of 0.6 N phosphoric acid and acetone (5:13) were added and shaken vigorously for a few seconds and centrifuged at 3000 g for 15 min. The absorbance of the supernatant was measured at 620 nm and the result was expressed as µg Evans blue dye/g of tissue [43].

#### 2.10.6. Determination of malondialdehyde (MDA) in gastric mucosa

The content of malondialdehyde was estimated in each supernatant by thiobarbituric reaction, as described by Ohkawa et al. [44]. To 1 ml gastric mucosal homogenates, were added 0.5 ml of 30% trichloroacetic acid followed by 0.5 ml of 0.8% thiobarbituric acid reagent. The reactive mixture after incubation for 30 min in boiling water was cooled and centrifuged at 3000 g for 15 min at 4 °C. Then absorbance of the supernatant was measured at 540 nm against reagent blank and the amount of MDA (nmol MDA/mg protein) was calculated.

#### 2.10.7. Determination of superoxide dismutase (SOD) activity

Superoxide dismutase *(SOD)* was measured by the pyrogallol oxidation inhibition assay of Marklund and Marklund [45] and the results were expressed as units (U) of SOD activity/mg protein. Briefly, the reaction mixture consist of 0.1 ml of supernatant, 25 µl of pyrogallol solution in 10 mM HCl, and volume was made up to 3 ml with tris-HCl buffer. The change in absorbance at 420 nm was recorded at 1 min interval for 3 min. A unit of SOD was defined as an amount of enzyme that causes 50% inhibition of pyrogallol auto-oxidation.

### 2.11. Histological evaluation of gastric lesions

Histological evaluation of stomach tissue from treated and untreated rats was carried out using 10% formaldehyde for the fixation. The samples were embedded in paraffin followed by the 5 µm sectioning and staining with haematoxylin and eosin solution. The specimens were observed under Olympus digital microscope (Olympus Corporation, Tokyo, Japan) assisted with a 1/3″ CCD Sony camera.

### 2.12. Statistical analysis

Data were represented as mean ± S.E.M. for six rats. Analysis of variance (ANOVA) was followed by Tukey’s post-test using GraphPad Prism software version 4.01 (GraphPad Software, Inc., San Diego, CA, USA) for the determination of the level of significance. Values of *p* < .05 were considered statistically significant.

## 3. Results

### 3.1. Phytochemical screening of GCE

**Figure 1.**
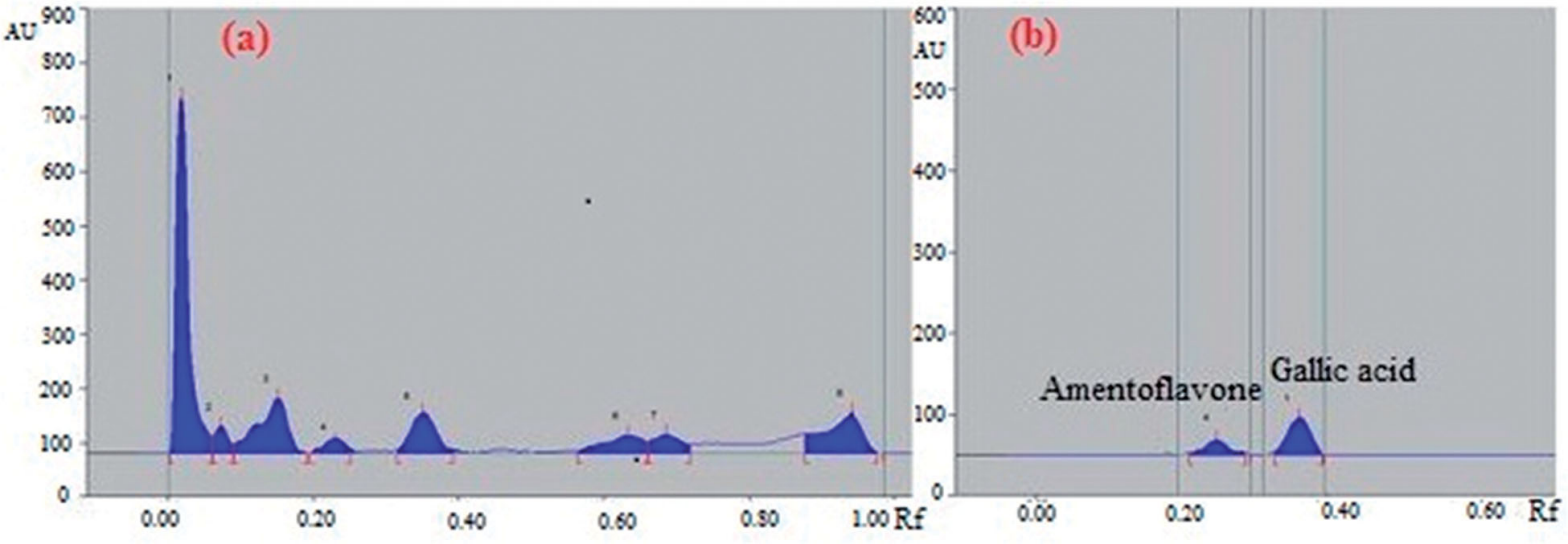
(a) HPTLC fingerprint chromatogram of GCE under UV 254 nm. (b) HPTLC chromatogram of amentoflavone and gallic acid present in GCE scanned at 366 nm.

Preliminary phytochemical analysis of GCE confirmed the presence of carbohydrate, glycoside, saponins, proteins, and polyphenolic constituents like flavonoids as major constituents in the extract. The preliminary HPTLC studies revealed that solvent system toulene: ethyl acetate: formic acid (8:2:1) was ideal for the GCE and gave eight well-resolved peaks with different R_f_ values (0.06, 0.09, 0.19, 0.25, 0.39, 0.66, 0.72, and 0.98) at a wavelength 254 nm which can be used as identifying marker (Figure 1(a)). The quantitative HPTLC determination showed the presence of 0.27%, 0.11% w/w of gallic acid and amentoflavone, respectively, in GCE (Figure 1(b)).

### 3.2. Characterization of bioactive compounds by HPLC

**Figure 2.**
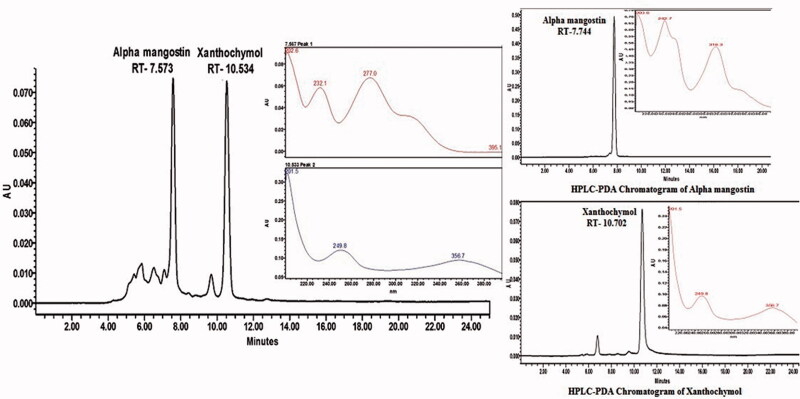
HPLC-PDA chromatogram of GCE and standard alpha mangostin and xanthochymol.

HPLC-PDA chromatogram of standard α-mangostin, xanthochymol, and of the *G. cowa* extract has been shown in Figure 2. The content of α-mangostin and xanthochymol in the *G. cowa* extract sample was 0.72 and 8.46%, respectively.

### 3.3. Total phenolic and flavonoid content of GCE

The total phenolic and flavonoid content of GCE was estimated to be 64.72 ± 1.13 mg gallic acid equivalents/g of dry extract, and 56.18 ± 0.84 mg rutin equivalents/g of dry extract from triplicate measurements, respectively.

### 3.4. Acute oral toxicities studies of GCE

From the acute toxicity study, it was observed that GCE at tested doses up to 5000 mg/kg, p.o. did not produce any symptom of toxicity or mortality on the experimental animals. To optimize the dose levels, 1/10th (200 mg/kg body weight) and 1/5th (400 mg/kg body weight) of the minimum effective dose (2000 mg/kg b.w.) was given for acute toxicity study were selected for the evaluation.

### 3.5. Effect of GCE on ethanol-induced gastric lesion and gastric pH

**Figure 3.**
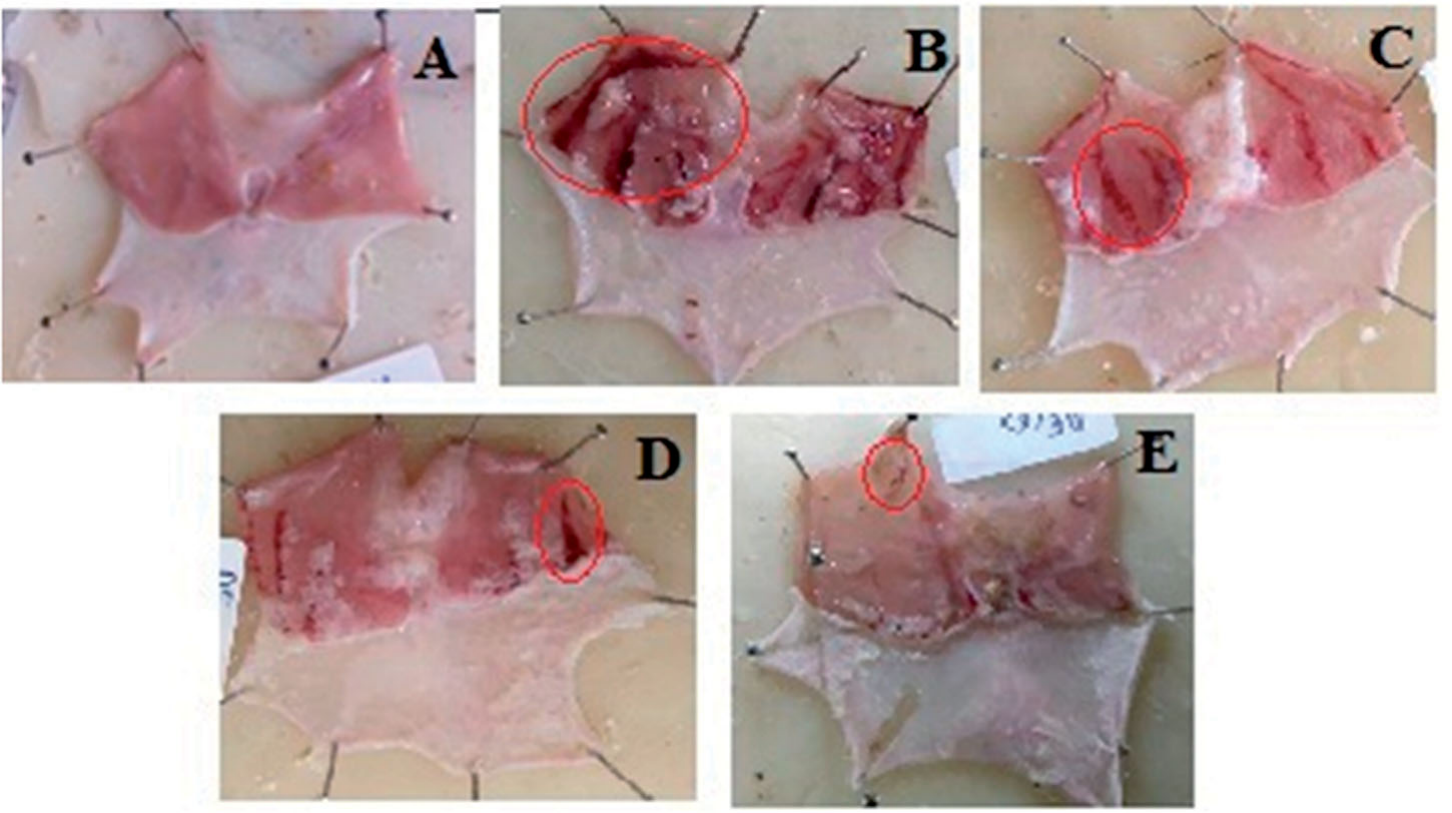
Effect of GCE on the macroscopic appearance of the stomach mucosa in ethanol-induced stomach mucosa injuries in rats. (A) Normal control group exhibited no injuries to the gastric mucosa. (B) Ulcer control group had severe injuries to stomach mucosa. (C) 200 mg/kg and (D) 400 mg/kg doses of GCE had moderate to mild disruption of surface epithelium in the gastric mucosa in dose-dependent manner. (E) Ranitidine showed mild disruption of surface epithelium in gastric mucosa. Red circle points to the haemorrhagic bands.

The protective effect of pre-treatment with GCE on ethanol-induced gastric lesions was evaluated (Table 2). Oral pre-treatment with GCE graded dose attenuated the number and severity of gastric lesions in a dose-dependent way. The gross appearance of the stomach of the normal control group showing its normal appearance and no macroscopic lesions were found (Figure 3(A)). In the ulcer control group, severe gastric lesions were observed in the mucosa layer, such as haemorrhagic streak (Figure 3(B)), with an ulcer index of 27.03 ± 0.82. Pre-treatment with GCE at the dose level of 200–400 mg/kg, and RAN (50 mg/kg) (Figures 3(C–E), respectively) significantly decreased the ulcer index compared to ulcer control, with a value of 21.91 ± 0.98, 15.13 ± 0.56, and 12.61 ± 0.73, respectively. The percentage protection of GCE at a higher dose level was 44.02%. As shown in Table 2, the level of pH was found to be decreased by 1.3-fold in the ethanol-treated rats when compared with the control group. However, animals pre-treated with GCE at the dose level of 200–400 mg/kg and RAN (50 mg/kg) significantly increased the pH values in a dose-dependent manner with maximum increase produced by ranitidine (1.6-fold).

**Table 2.** Effect of GCE and ranitidine on ethanol-induced ulcers in experimental rats.

Treatment (mg/kg)	Ulcer index	% Protection	pH of gastric content
Normal control	0.00		3.731 ± 0.15
Ulcer control	27.03 ± 0.82	–	2.87 ± 0.13^#^
GCE 200	21.91 ± 0.98^b^	18.94	3.63 ± 0.12^b^
GCE 400	15.13 ± 0.56^c^	44.02	3.92 ± 0.09^c^
RAN 50	12.61±.73^c^	52.12	4.64 ± 0.09^c^

Values are expressed as means ± S.E.M. (*n* = 6 in each group). Statistical comparison was analysed by a one-way ANOVA followed by Tukey’s multiple comparison tests.

^#^*p* < .001 compared to the respective control group.

^b^*p* < .01, ^c^*p* < .001 statistically significant in comparison with the ulcer control group.

### 3.6. Effect of GCE on gastric wall mucus level in gastric tissue

**Figure 4.**
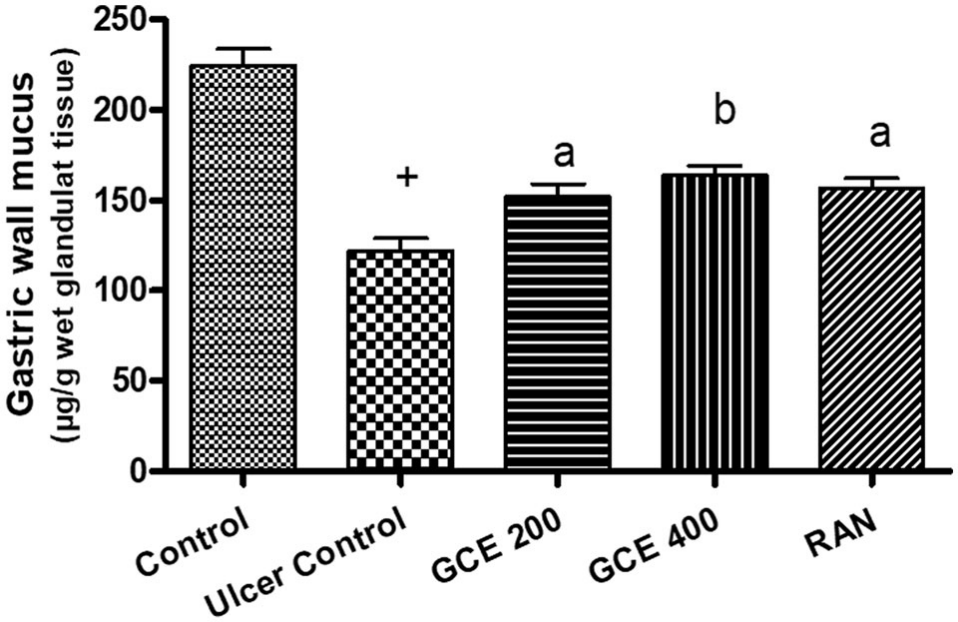
Effect of GCE on gastric wall mucus in the EtOH-induced ulcer group. Values are expressed as means ± S.E.M. (*n* = 6 in each group); ^+^*p* < .001 compared to the respective control group. ^a^*p* < .05, ^b^*p* < .01 compared to the respective EtOH-induced ulcer control group.

As shown in Figure 4, that ethanol administration significantly decreased the gastric wall mucus to 122.07 ± 7.08 (*p* < .001), but administration of GCE at 200–400 mg/kg dose level significantly (*p* < .01) enhanced the gastric wall mucus to 152.26 ± 6.84–163.69 ± 5.31, respectively (24.73–34.09%).

### 3.7. Effect of GCE on gastric non-protein sulfhydryls level in gastric tissue

The result showed that ethanol significantly decreased the gastric mucosal NP-SH content compared to the normal control group (*p* < .001). However, this decrease in NP-SH level was significantly increased by a higher dose (400 mg/kg) of GCE. Although, the level of NP-SH contents was also improved by a lower dose (200 mg/kg) of GCE but it was not found to be statistically significant (Table 3).

**Table 3.** Effect of GCE on the level of non-protein sulfhydryl (NP-SH) in gastric mucosa of rats treated with ethanol.

Treatment (mg/kg)	Non-protein sulfhydryl (µmol/g of tissue)
Control	2.497 ± 0.13
Ulcer control	1.511 ± 0.09^#^
GCE 200	1.743 ± 0.06
GCE 400	2.015 ± 0.05^b^
RAN	2.053 ± 0.10^b^

Values are expressed as means ± S.E.M. (*n* = 6 in each group).

^#^*p* < .001 compared to the respective control group.

^b^*p* < .01 compared to the respective EtOH-induced ulcer group.

### 3.8. Effect of GCE on mucosal microvascular permeability

**Figure 5.**
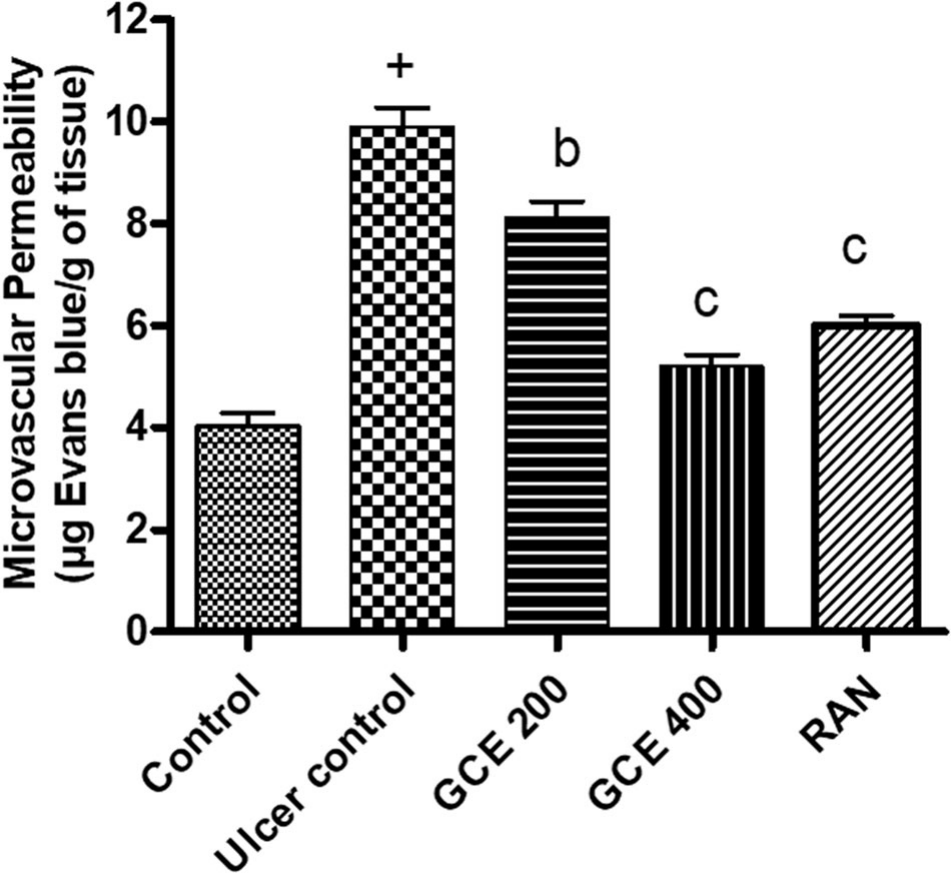
Effect of GCE on microvascular permeability (extravasation of Evans blue) in gastric mucosa induced by absolute ethanol. Values are expressed as means ± S.E.M. (*n* = 6 in each group); ^+^*p* < .001 compared to the respective control group. ^b^*p* < .01 and ^c^*p* < .001 compared to the respective ethanol-induced ulcer control group.

Pre-treatment with a graded dose of GCE (200 and 400 mg/kg) and standard drug ranitidine (50 mg/kg) dose-dependently attenuated ethanol-induced increased vascular permeability in gastric mucosa by 17.91, 47.43, and 39.29%, respectively (Figure 5).

### 3.9. Effect of GCE on malondialdehyde level in gastric tissue

**Figure 6.**
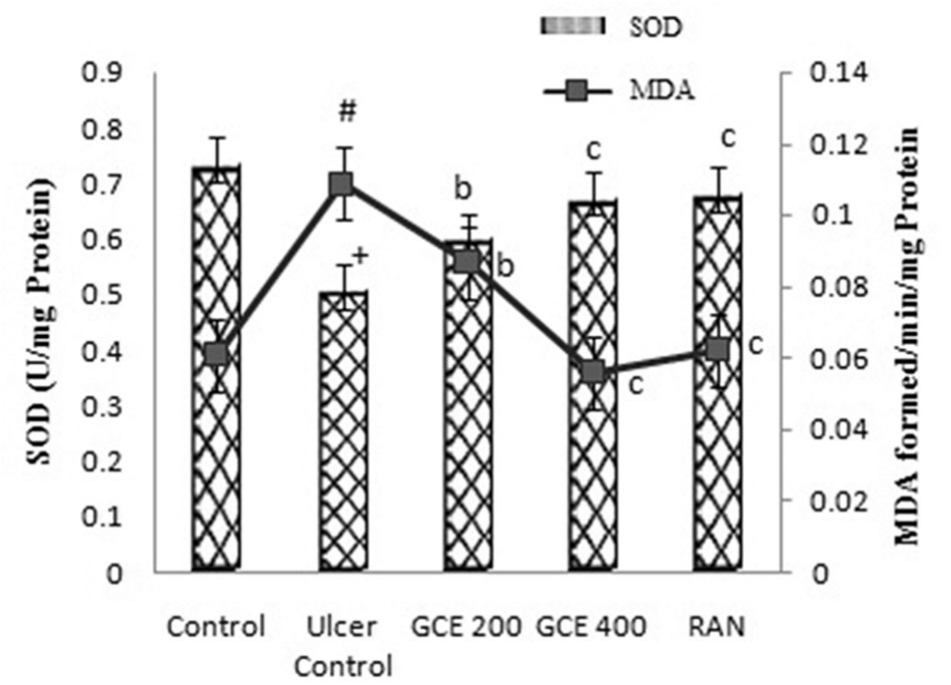
Effect of GCE on MDA and SOD activity in rat gastric mucosa in ethanol-induced gastric ulcers in rats. Values are expressed as means ± S.E.M. (*n* = 6 in each group); ^+^*p* < .001, ^#^*p* < .01 compared to the respective control group. ^b^*p* < .01 and ^c^*p* < .001 compared to the respective ethanol-induced ulcer control group.

As shown in Figure 6, ethanol increased the gastric MDA level (44%) in the ulcer control group when compared with the normal control group significantly (*p* < .001). Rat pre-treated with GCE at the dose of 200 and 400 mg/kg, similar to the ranitidine group (50 mg/kg), exhibited a significant reduction in MDA level in stomach tissue (*p* < .01).

### 3.10. Effect of GCE on superoxide dismutase level in gastric tissue

The level of SOD (30.72%) in gastric tissue of rodents was significantly decreased in the ethanol-induced ulcer control group when compared with the normal control group. A significant increase (*p* < .001) in the levels of SOD was observed in rats pre-treated with a higher dose (400 mg/kg) of GCE. RAN at the dose of 50 mg/kg significantly increased the SOD value (*p* < .001) (Figure 6).

### 3.11. Effect of GCE on histopathology of stomach

**Figure 7.**
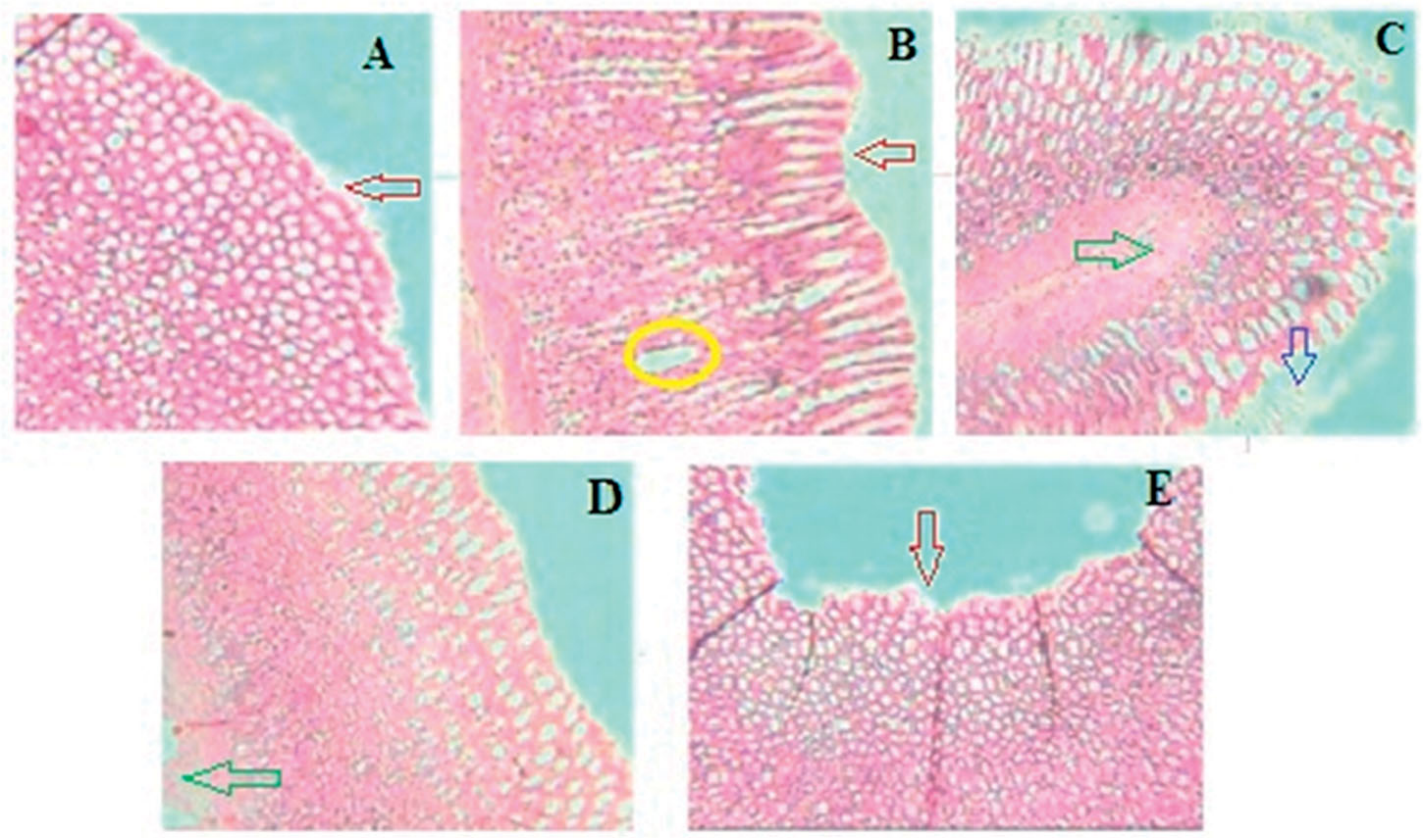
Effect of GCE on the histology of ethanol-induced stomach mucosal injury. (A) Normal control group showing intact mucosal lining with flattened epithelial cells and no lesions. (B) Ulcer control group showing severe disruption of surface epithelium (red arrow) and haemorrhagic necrotic longitudinal lesion (yellow circle) and edoema of submucosal layer. The animal pre-treated with GCE in (C) 200 mg/kg and (D) 400 mg/kg groups shows moderate to mild disruption of surface epithelium; reduction in submucosal edoema (green arrow) and surface epithelium is partially restored in a dose-dependent manner. (E) Ranitidine showing mild disruption of the surface epithelium (red arrow), deep mucosal necrosis is absent but mucosal edoema persists.

Histopathological observation showed severe damage in the gastric mucosa characterized by extensive disruption of the surface epithelium, necrotic lesion in the mucosa, extensive edoema of the submucosa, and leukocyte infiltration in the mucosal layer of ethanol-treated rats. Whereas rats pre-treated with GCE graded doses produced mild mucosal damage, leukocyte infiltration, and mucosal injury (Figure 7).

## 4. Discussion

Ethanol-induced gastric ulcer is one of the well-established experimental models to investigate the new ulcer protective drugs. Administration of ethanol to the fasted experimental animals resulted in gastric mucosal damage leading to the formation of haemorrhagic streak in the glandular part of the stomach (Figure 3(B)) and an increase in ulcer index. The gastric mucosal lesion caused by ethanol is associated with a reduction in gastric blood flow, vascular permeability, and depletion of gastric mucus content [46]. In addition, ethanol induces oxidative stress by enhancing the production of malondedialdehyde and reducing glutathione production [47]. Furthermore, the production of reactive oxygen species (ROS) by ethanol is likely to play a significant role in ulcer formation [48]. As a result, ethanol was chosen to cause stomach ulcers in rats in our investigation.

Our finding showed that pre-treatment with GCE reduced the number and size of the gastric lesion in mucosa induced by ethanol (Figures 3(C,D)) and simultaneously replenished the depleted levels of gastric wall mucus (Figure 4). It can be predicted that GCE effectively suppresses the destruction of gastric wall mucus and also suppresses gastric acidity in comparison with the ulcer control group. This result was further confirmed by the beneficial effect of GCE on the NP-SH content of gastric mucosa in stomach tissue, indicating that GCE has a protective effect through reinforcing the gastric mucosa. NP-SH plays an important function in tissue protection by scavenging oxidant products. According to Szabo and Vattay [49], the endogenous NP-SH complex is a major component in mucosal protection against ethanol-induced stomach damage. Furthermore, GCE was shown to decrease the permeability of dye produced by ethanol in the stomach mucosal vascular system. Increased vascular permeability appears to be the rate-limiting step in mucosal injury [50] and so reducing the rise in vascular permeability of stomach tissue may play an important role in mucosal protection.

In this context, it is feasible to speculate that *G. cowa* reduces the gastric lesions by protecting the adhered gastric mucus possibly due to the presence of polyphenolic compounds including gallic acid and amentoflavone in GCE and its potent antioxidant activity. The quantitative phytochemical analysis confirmed the presence of a high amount of phenolic and flavonoid content in GCE. The presence of gallic acid in the extract is the first time reported and justifies the potent antioxidant activity of the plant. Gallic acid scavenges the free radicals, block ^•^OH-mediated oxidative damage and plays an important role in the prevention and therapy of diseases including ulcer. Gallic acid has been reported to inhibit the lipid peroxidation of gastric cells and increase the synthesis of mucus in chemical and physical induced ulcer model [51]. Amentoflavone has been also reported to play an important role in the treatment of gastric ulcer by protecting the mucosal damage through reducing vascular permeability [52]. Two other biologically important compounds α mangostin and xanthochymol present in the extract were also characterized by HPLC which have been reported to have potent antioxidant and protective effects [21]. Hence, the polyphenolic compounds (gallic acid and amentoflavone) and xanthone (α mangostin) were chosen for the standardization of the *G. cowa* extract. However, the chemical constituents of extract responsible for activity are not known but many phenolic and flavonoid compounds and other bioactive components like saponins, sterols (β-sitosterol) present in the extract have been reported to possess antiulcer activity [53,54].

It is well-known that reactive oxygen species (ROS) are among most the important factors involved in the pathogenesis of ethanol-induced gastric lesions mediated by oxidative stress [55]. Several studies have shown that potent antioxidant and free radical scavengers inhibit oxidative stress and consequently the progression of lipid peroxidation [56,57]. Compounds with this capability, such as phenolic, flavonoid compound, and ascorbic acid are reported to be present in the fruit of *G. cowa* [58]. Therefore, pre-treatment with GCE in this study probably improves cellular antioxidant defense. In agreement with previous studies, the current findings showed that intragastric administration of ethanol in rats led to a significant increase in the level of MDA in gastric tissue. MDA is commonly measured as a biomarker to assess the level of lipid peroxidation in tissue [59]. Pre-treatment with GCE (200 and 400 mg/kg) exhibited antioxidant properties by decreasing the MDA level, suggesting its potentials to protect lipid peroxidation induced by ethanol in rats. Furthermore, GCE restored the depleted level of the antioxidant activity of the SOD enzyme after ethanol administration, thus protecting the gastric mucosal lesion. These results suggest the possible involvement of endogenous antioxidants in the experimental protective effect of *G. cowa* fruit extract in the gastric lesion.

The stomachs were also examined histopathologically to corroborate the findings of the present investigation. According to histological examination, ethanol treatment caused haemorrhagic necrosis in the gastrointestinal mucosa of rats. Pre-treatment with GCE resulted in minimum ulceration or haemorrhage in the gastric antrum. The results reported in pharmacological and biochemical parameters are supported by these findings. Sub-acute toxicity study illustrated that GCE to be a safe drug up to 5000 mg/kg, p.o. The human equivalency dose of GCE was calculated according to the formula for dose translation based on body surface area [60]. Considering the average human body weight of 60 kg, the estimated human daily dose of GCE would be 3.891 gm/day. The estimated human daily dose may be used for clinical study of the *G. cowa* extract.

According to the findings, GCE in comparison to the conventional antiulcer therapies may be a better alternative treatment, since herbal drugs including GCE have fewer side effects, are less expensive, and can scavenge free radicals. While conventional treatments are associated with various side effects including relapse of the disease as described in Table 1 and are often expensive for the poor rural populations [61]. Numerous studies have shown that the efficacy of herbal remedies is similar to or better than that of drugs like ranitidine or omeprazole in humans and animal models [10,12]. This was further confirmed through the present study carried out, where the efficacy of GCE for the reduction in gastric lesions was comparable to standard antiulcer drug ranitidine. Toxicity studies on *Garcinia* showed that it is safe for human ingestion, with a wide margin of safety [62]. Although herb-drug interactions have raised safety concerns and some herbs can cause side effects [63] but according to the available literature, no interactions of *Garcinia* with other co-administered drugs have been reported so far.

In conclusion, the findings of the present study showed that GCE has a significant protective effect against gastric ulcers in rats, which may be due to its ability to improve the mucosal defence barrier and provide antioxidant protection against oxidative stress-induced gastric damage. These findings back up the traditional uses of *G. cowa* and add to its pharmacological validity. The exploration of the underlying mechanisms responsible for the pharmacological activity of *G. cowa* will be the focus of our future research.

## Data Availability

All data generated within this study are available from the corresponding author on request.
